# Supplementary material for: A global meta-analysis on the effects of organic and inorganic fertilization on grasslands and croplands
Source: Nat Commun. 2024 Apr 22;15:3411. doi: 10.1038/s41467-024-47829-w (PMC11035549; doi:10.1038/s41467-024-47829-w)
Supplement: Supplementary file 1 — Supplementary Information [file 41467_2024_47829_MOESM1_ESM.pdf]

Supplementary Information

Supplementary Figure 1. Flow diagram showing the process of literature search in this study.

Supplementary Figure 2. The geographic distribution of all study sites in this meta-analysis.

Supplementary Figure 3. Funnel plots of potential publication bias of aboveground biomass, species richness, Pielou evenness index and soil organic carbon datasets.

Supplementary Figure 4. The importance of environmental predictors of aboveground biomass and species richness response ratio under nutrient addition.

Supplementary Figure 5. Structural equation model testing the effects of environment factors on aboveground biomass and species richness following nutrient addition.

Supplementary Figure 6. The responses of soil organic carbon to organic fertilization across environmental gradients in global grasslands and croplands.

Supplementary Table 1. Egger's regressions of aboveground biomass, species richness, Pielou evenness index and soil organic carbon.

Supplementary Table 2. Values of weighted mean response ratio (ln RR++) and corresponding confidence intervals in the meta-analysis as shown in Figure 1 in the main text.

Supplementary Table 3. Description of environmental factors.

Supplementary Table 4. Model selection results for the effect of aboveground biomass response ratio under organic fertilization.

Supplementary Table 5. Model selection results for the effect of species richness response ratio under organic fertilization.

Supplementary Table 6. Model selection results for the effect of soil organic carbon response ratio under organic fertilization.

Supplementary Table 7. Model preselection results for the effect of aboveground biomass response ratio under inorganic fertilization.

Supplementary Table 8. Model preselection results for the effect of species richness response ratio under inorganic fertilization.

Supplementary Table 9. Coefficients estimated in the linear mixed model for aboveground biomass, species richness and soil organic carbon response ratio under organic fertilization.

Supplementary Table 10. Coefficients estimated in the linear mixed model for aboveground biomass and species richness response ratio under inorganic fertilization.

Supplementary Table 11. The coefficients of the linear mixed models for Figure 4**a–b**.

Supplementary Table 12. The path coefficients of structural equation models for organic and inorganic fertilization.

Supplementary Table 13. The test statistics of the Standardised Major Axis Tests and Routines (SMATR) with ordinary least squares regression technique for Figure 5**c–e**.

Supplementary Note. PRISMA checklist.

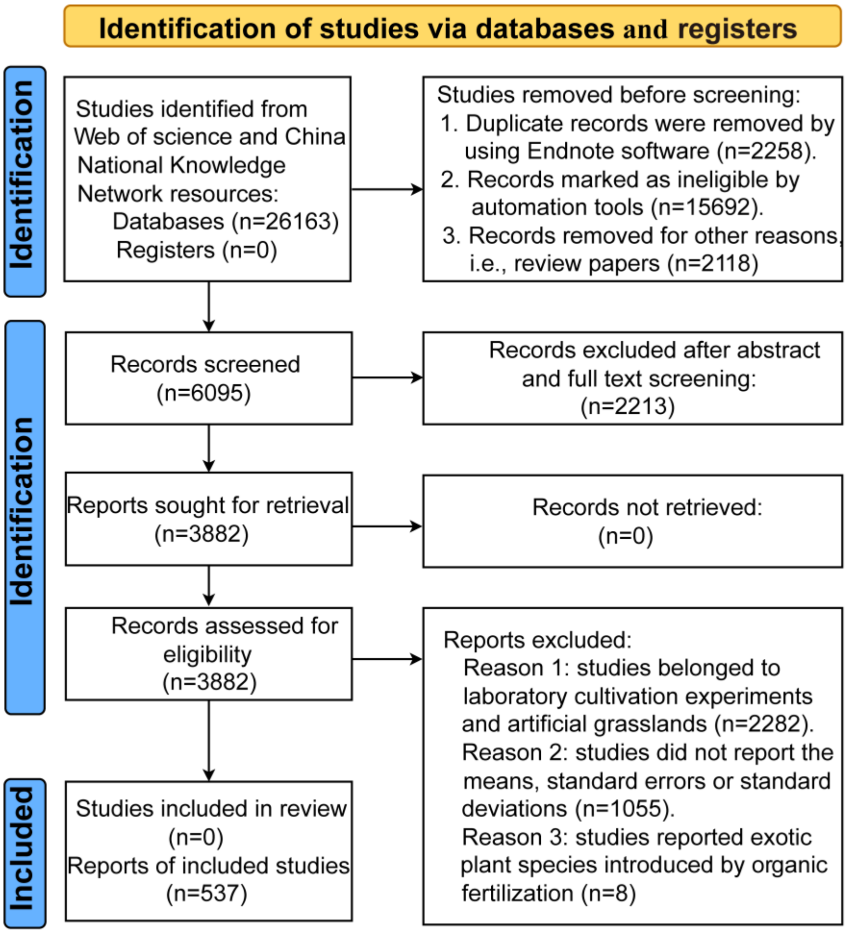

Supplementary Figure 1. Flow diagram showing the process of literature search in this study.

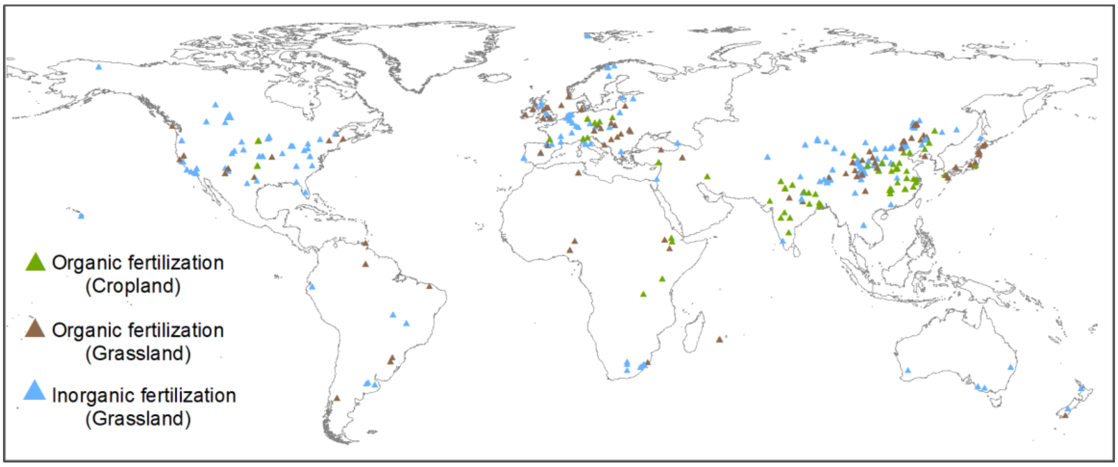

Supplementary Figure 2. The geographic distribution of all study sites in this meta-analysis. The map was created with ArcGIS version 10.8 (ESRI, USA). Global map was downloaded from natural earth (https://www.naturalearthdata.com/). Source data are provided as a Source Data file.

Supplementary Table 1. Egger's regressions of aboveground biomass, species richness, Pielou evenness index and soil organic carbon. The table reports two-sided *p*-values (null hypothesis: slope=0).

Response variables	Egger regression		
	Treatment	Z	*p*
Aboveground biomass	Inorganic	2.74	0.006
Aboveground biomass	Organic	0.67	0.504
Species richness	Inorganic	−3.75	0.0002
Species richness	Organic	2.54	0.011
Pielou evenness index	Inorganic	−3.39	0.0007
Pielou evenness index	Organic	1.75	0.079
Soil organic carbon	Inorganic	3.70	0.0002
Soil organic carbon	Organic	1.12	0.261

Note: Z is the egger regression intercept; $p>0.05$ indicates that the result is robust without potential publication bias (Egger's test); $p<0.05$ indicates a potential publication bias. Source data are provided as a Source Data file.

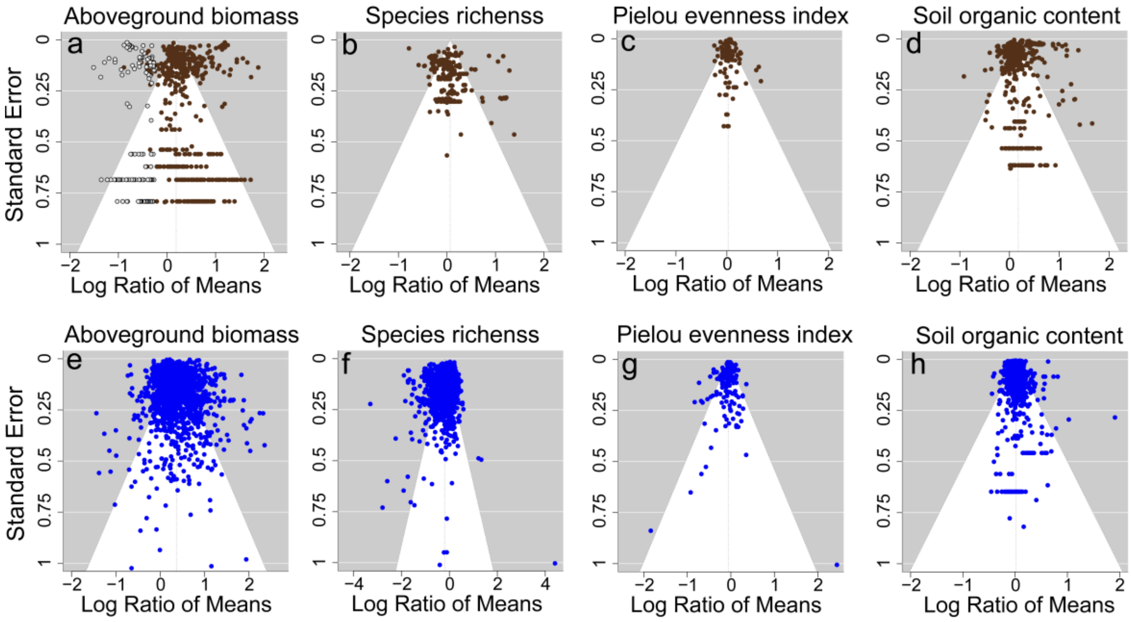

Supplementary Figure 3. Funnel plots of potential publication bias of aboveground biomass, species richness, Pielou evenness index and soil organic carbon datasets. Panels **a**, **b**, **c**, and **d** represent organic fertilization. Panels **e**, **f**, **g** and **h** represent inorganic fertilization. Source data are provided as a Source Data file.

Egger's regression suggested that there was potential publication bias in aboveground biomass, species richness, Pielou evenness index and soil organic carbon datasets under inorganic fertilization (Supplementary Table 1), while the trim and fill analysis suggested that there were no missing values (Supplementary Figs. 2**e**, **f**, **g** and **h**). In addition, we found a potential publication bias in aboveground biomass and species richness datasets under organic fertilization, and the trim and fill results are shown in Supplementary Figure 2**a**–**b**. The trim and fill analysis suggested that there was no missing value in species richness under organic fertilization (Supplementary Fig. 2**b**). There were some missing values for aboveground biomass, but we found that the meta-analysis results for aboveground biomass had little impact before and after trimming and filling (before: ln RR++ = 0.37, $p < 0.001$; after: ln RR++ = 0.19, $p < 0.001$). Therefore, our meta-analysis results were robust and reliable.

Supplementary Table 2. Values of weighted mean response ratio (ln RR++) and corresponding confidence intervals in the meta-analysis as shown in Figure 2 in the main text. Source data are provided as a Source Data file. The table reports two-sided *p*-values.

Response variables	Treatment	ln RR++	Percent change (%)	*p* value	95%CIs	Sample sizes
Aboveground biomass	Inorg vs AMB	0.3385	42.03	**<0.001**	(39.95, 44.12)	1540
	Org vs AMB	0.4423	55.63	**<0.001**	(50.61, 60.65)	350
	Org vs Inorg	0.0529	5.43	0.138	(−1.81, 12.67)	110
Species richness	Inorg vs AMB	−0.1982	−17.98	**<0.001**	(−19.57, −16.39)	1625
	Org vs AMB	0.0386	3.93	0.181	(−1.88,9.75)	155
	Org vs Inorg	0.0978	10.27	**<0.001**	(5.00, 15.95)	71
Pielou evenness index	Inorg vs AMB	−0.0618	−5.99	**<0.001**	(−8.27, −3.72)	191
	Org vs AMB	0.0002	0.02	0.986	(−2.05, 2.01)	89
	Org vs Inorg	0.0925	9.69	**<0.001**	(5.36, 14.02)	38
Soil organic carbon	Inorg vs AMB	0.0197	1.99	**<0.001**	(0.96, 3.01)	799
	Org vs AMB	0.1710	18.65	**<0.001**	(15.57, 21.73)	388
	Org vs Inorg	0.1419	15.25	**<0.001**	(9.20, 21.29)	21

Note: Inorganic fertilization, Inorg; organic fertilization, Org; ambient conditions, AMB. Value in bold indicates a significant effect ($p < 0.05$).

To select the set of environmental factors that best predicted the response pattern of biomass, plant diversity and soil organic carbon to nutrient addition, we conducted a multi-model inference procedure using the dredge function in R "*MuMIn*" package. We conducted a complete predicted model for aboveground biomass, species richness and soil organic carbon with site as a random effect and including all environmental factors as shown in Table 3.

The complete models were:

lmer (Aboveground biomass response ratio~ MAT + pH + SOCD + SBD + SWC + SCEC + Sand + TN + Org + N + P + K+ Niche + (1|site), data = data, REML ="TRUE")

lmer (Species richness response ratio ~ MAT + pH + SOCD + SBD + SWC + SCEC + Sand + TN + Org + N + P + K+ Niche + (1|site), data = data, REML =" TRUE ")

lmer (soil organic carbon response ratio ~ MAT + pH + SBD + SWC + SCEC + Sand + TN + Org +Initial SOC+ experimental duration (1|site), data = data, REML =" TRUE ")

Supplementary Table 3. Description of environmental factors.

Environmental factors	Unit	Key
Mean average temperature	°C	MAT
Soil pH	—	pH
Soil organic carbon density	kg m^{-3}	SOCD
Soil bulk density	g cm^{-3}	SBD
Soil water content	%	SWC
Soil cation change capacity	cmol [+] kg^{-1}	SCEC
Soil sand content	%	Sand
Soil total nitrogen	g kg^{-1}	TN
Organic fertilizer amount added	kg m^{-2}	Org
Nitrogen fertilizer rate	g m^{-2}	N
Phosphorus fertilizer rate	g m^{-2}	P
Potassium fertilizer rate	g m^{-2}	K
Number of nutrients added	—	Niche

The best models selected for organic fertilization are shown in Tables S4–6. The first row in Tables S4–6 suggested the best models with the lowest Akaike Information Criterion (AIC). Source data are provided as a Source Data file.

Supplementary Table 4. Model selection results for the effect of aboveground biomass response ratio under organic fertilization. See Table 3 for environmental factors.

Models	Model selection table									
	MAT	N	Niche	Org	P	pH	Sand	SCEC	TN	AIC
Model1	0.4309	0.1540		0.3712	0.1472				0.4111	294.2
Model2	0.4540	0.1528		0.3720	0.1411			0.1601	0.3513	296.8
Model3	0.4130		0.1798	0.3468					0.3977	297.4
Model4	0.5223	0.1565		0.3751	0.1438	0.1434			0.5144	297.4
Model5	0.4111	0.1639		0.3712					0.4003	298.3
Model6	0.4200	0.1525		0.3672	0.1408		−0.0890		0.4067	298.4

Supplementary Table 5. Model selection results for the effect of species richness response ratio under organic fertilization. See Table 3 for environmental factors.

Models	Model selection table					
	P	SBD	SCEC	SOCD	SWC	AIC
Model1		0.8508	0.3932		0.4798	139.0
Model2		0.4892	0.3779			139.9
Model3		0.9448		−0.3523	0.4740	140.2
Model4	0.2112	0.5066	0.3743			140.3
Model5	0.1892	0.8243	0.3896		0.4224	140.4
Model6		0.5825		−0.3329		140.8

Supplementary Table 6. Model preselection results for the effect of soil organic carbon response ratio under organic fertilization. See Table 3 for environmental factors.

Models	Model selection table (Grassland)								
	Initial SOC	Org	MAT	pH	Sand	SBD	SCEC	TN	AIC
Model1	−0.1494	0.1173	0.3165		0.1576				797.6
Model2	−0.1860	0.1163	0.3708		0.1402			0.1146	798.1
Model3	−0.1607	0.1145	0.4674	0.1712	0.1417			0.1712	798.1
Model4	−0.1849	0.1195	0.3731		0.1463	0.1096		0.1847	799.1
Model5	−0.1275	0.1173	0.3508	0.0857	0.1630				799.1
Model6	−0.1610	0.1178	0.3549		0.1649		0.0657		799.2
Models	Model selection table (Cropland)								
	Initial SOC	Org	MAT	pH	Sand	SBD	SCEC	TN	AIC
Model1	−0.3836	0.1646	−0.2279	−0.1869				−0.2082	954.1
Model2	−0.3668	0.1635	−0.2532	−0.2112		0.1046		−0.1960	954.7
Model3	−0.3988	0.1632	−0.2298	−0.1755	−0.0754			−0.2046	955.1
Model4	−0.4338	0.1577	−0.1307						955.4
Model5	−0.3824	0.1619	−0.2575	−0.2006	−0.0828	0.1135		−0.1910	955.5
Model6	−0.3936	0.1648	−0.2321	−0.1977			0.0527	−0.2186	955.7

The model selected results under inorganic fertilization are shown in Tables S7–8. Source data are provided as a Source Data file.

Supplementary Table 7. Model preselection results for the effect of aboveground biomass response ratio under inorganic fertilization. See Table 3 for environmental factors.

Models	Model selection table						
	N	Niche	Sand	SCEC	SOCD	SWC	AIC
Model1	0.151	0.424				0.153	1175.1
Model2	0.156	0.428					1175.4
Model3	0.168	0.390			0.137		1175.7
Model4	0.161	0.397			0.102	0.118	1178.1
Model5	0.149	0.427	0.096			0.169	1178.3
Model6	0.158	0.429		0.0864			1179.2

Supplementary Table 8. Model preselection results for the effect of species richness response ratio under inorganic fertilization. See Table 3 for environmental factors.

Models	Model selection table							
	N	Niche	P	Sand	SBD	SOCD	SWC	AIC
Model1	−0.404		−0.228					1261.8
Model2	−0.406		−0.221	0.1021				1264.6
Model3	−0.402		−0.226				−0.078	1265.9
Model4	−0.394	−0.062	−0.201					1266.2
Model5	−0.405		−0.225			−0.053		1266.9
Model6	−0.406		−0.227		0.044			1267.1

Supplementary Table 9. Coefficients estimated in the linear mixed model for aboveground biomass, species richness and soil organic carbon response ratio under organic fertilization.

lmer (Aboveground biomass response ratio ~ MAT + Org + TN + N + P + (1|site), data=data, REML =" TRUE ")					
Fixed effects:					
	Estimates	SE	df	t value	*p* value
(Intercept)	0.038	0.095	47.471	0.403	0.689
MAT	0.431	0.104	45.758	4.144	**<0.001**
Org	0.371	0.0615	140.143	6.035	**<0.001**
TN	0.411	0.103	45.361	3.991	**<0.001**
N	0.154	0.042	119.362	3.65	**<0.001**
P	0.143	0.0427	118.619	3.345	**0.001**
Marginal R^2 / Conditional R^2: 0.52 / 0.85					

lmer (Species richness response ratio ~ SBD + SWC + SCEC + (1|site), data = data, REML =" TRUE ")					
Fixed effects:					
	Estimates	SE	df	t value	*p* value
(Intercept)	0.0151	0.148	14.595	0.102	0.920
SBD	0.851	0.212	14.896	4.024	**0.001**
SWC	0.479	0.211	14.351	2.279	**0.038**
SCEC	0.393	0.139	17.207	2.81	**0.012**
Marginal R^2 / Conditional R^2: 0.48 / 0.73					

lmer (Soil organic carbon response ratio ~ Initial SOC + Org + MAT + Sand + (1|site), data = data, REML =" TRUE ") (Grassland)					
Fixed effects:					
	Estimates	SE	df	t value	*p* value
(Intercept)	0.027	0.106	37.699	0.260	0.796
Initial SOC	−0.149	0.058	293.924	−2.569	**0.011**
Org	0.117	0.054	272.315	2.174	**0.031**
MAT	0.316	0.094	45.689	3.375	**0.002**
Sand	0.158	0.068	2.32	2.320	**0.023**
Marginal R^2 / Conditional R^2: 0.20 / 0.63					

lmer (Soil organic carbon response ratio ~ Initial SOC + Org + MAT + TN+ pH +SBD + (1|site), data = data, REML =" TRUE ") (Cropland)					
Fixed effects:					
	Estimates	SE	df	t value	*p* value
(Intercept)	0.023	0.080	68.286	0.279	0.781
Initial SOC	−0.384	0.084	125.329	−4.548	**<0.001**
Org	0.164	0.060	301.465	2.723	**0.007**
MAT	−0.228	0.091	94.440	−2.505	**0.013**
TN	−0.208	0.101	111.549	−2.063	**0.041**

pH	−0.187	0.096	124.126	−1.956	**0.053**
Marginal R^2 / Conditional R^2: 0.16 / 0.44					

Note: Marginal R^2 and conditional R^2 indicate R^2 of fixed effects and random plus fixed effects, respectively. The values in bold indicate significant effects ($p < 0.05$). p value: Probability of type-I error (two-sided). See Table 3 for the environmental factors. Source data are provided as a Source Data file.

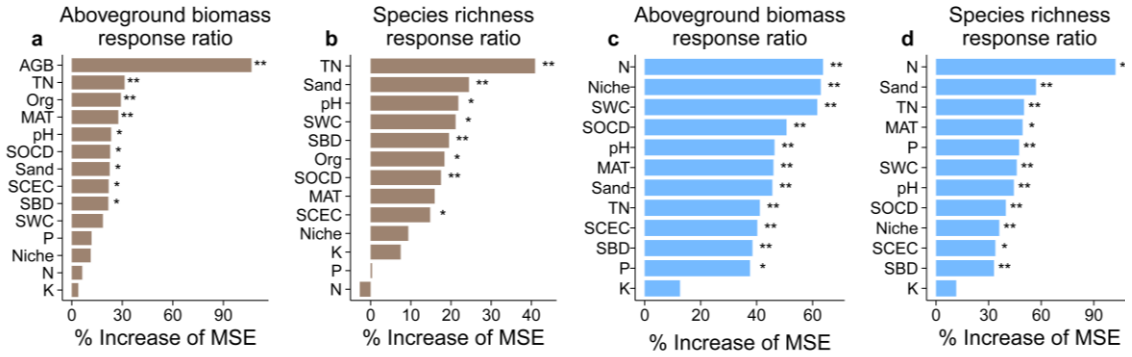

Supplementary Figure 4. The importance of environmental predictors of aboveground biomass and species richness response ratio under nutrient addition. Organic fertilization (**a**–**b**). Inorganic fertilization (**c**–**d**). **Key:** MSE: mean square error estimated by random forest model, MAT: mean annual temperature, TN: soil total nitrogen, Org: organic fertilizer amount added, Sand: soil sand content, pH: soil pH, SWC: soil water content, SOCD: soil organic carbon density, SBD: soil bulk density, SCEC: soil cation exchange capacity. N: nitrogen fertilizer rate, P: phosphorus fertilizer rate, K: potassium fertilizer rate, Niche: the number of nutrients added. Significance level: *$p < 0.05$ and **$p < 0.01$. Source data are provided as a Source Data file.

Supplementary Table 10. Coefficients estimated in the linear mixed model for aboveground biomass and species richness response ratio under inorganic fertilization.

lmer (Aboveground biomass response ratio ~ N + Niche + SWC + (1|site), data=data, REML =" TRUE ")

Fixed effects:					
	Estimates	SE	df	t value	*p* value
(Intercept)	−0.054	0.063	147.689	−0.864	0.389
N	0.151	0.035	446.759	4.311	**<0.001**
Niche	0.424	0.039	492.664	10.714	**<0.001**
SWC	0.153	0.062	141.547	2.484	**0.014**
Marginal R^2 / Conditional R^2: 0.23 / 0.66					

lmer (Species richness response ratio ~ N + P + (1|site), data = data, REML ="TRUE")

Fixed effects:					
	Estimates	SE	df	t value	*p* value
(Intercept)	−0.008	0.059	180.664	−0.135	0.893
N	−0.404	0.034	492.929	−11.869	**<0.001**
P	−0.228	0.041	525.242	−5.578	**<0.001**
Marginal R^2 / Conditional R^2: 0.21 / 0.63					

Note: See Table 3 for the environmental factors and see Table 8 for other details. *p* value: Probability of type-I error (two-sided). Source data are provided as a Source Data file.

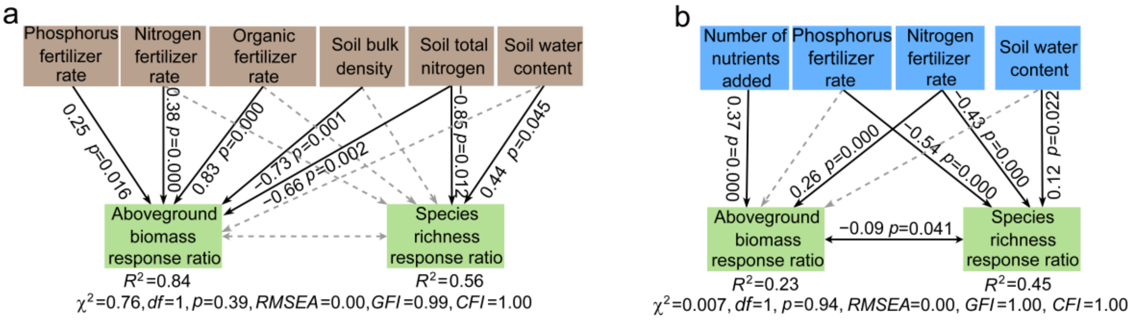

Supplementary Figure 5. Structural equation model testing the effects of environment factors on aboveground biomass and species richness following nutrient addition. Field experiments that measured both aboveground biomass and species richness were used in these analyses**.** (**a**) Organic fertilization. (**b**) Inorganic fertilization. We used structural equation models (SEM) to explore the direct and indirect effects of biotic and abiotic factors on the response of species richness to nutrient addition. The black solid and gray dashed arrows show significant (two-sided,

$p < 0.05$) and nonsignificant effects (two-sided, $p > 0.05$), respectively. The bidirectional arrow shows the covariances between variables. The standard path coefficients and significant *p*-value of the model are shown adjacent to arrows. Non-significant coefficients are not shown for simplicity. R^2 represents the proportion of variance explained for the dependent variable (**a**, **b**). When the number of nutrients added (Niche) was included in the SEM, the fitting coefficient test failed, therefore it was not included in the final model. Complete model statistical results were presented in Tables 12. Source data are provided as a Source Data file.

Supplementary Table 11. The coefficients of the linear mixed models for Figure 4a.

lmer (Species richness response ratio ~ Aboveground biomass response ratio + (1|site), data=data) (Organic fertilization)					
Predictor	Estimates	SE	df	t	*p* value
Intercept	−0.0031	0.1465	15.31	0.021	0.983
biomass	−0.1050	0.2045	22.78	−0.513	0.613
Marginal R^2 / Conditional R^2: 0.009 / 0.59					

Note: Marginal R^2 and conditional R^2 indicate R^2 of fixed effects and random plus fixed effects, respectively. The values in bold indicate a significant effect ($p < 0.05$). *p* value: Probability of type-I error (two-sided). Source data are provided as a Source Data file.

Table 11 (continued). The coefficients of the linear mixed models for Figure 4**b**.

lmer (Species richness response ratio ~ Aboveground biomass response ratio + (1|site), data=data) (Inorganic fertilization)					
Predictor	Estimates	SE	df	t value	*p* value
Intercept	−0.1281	0.0431	138.68	−2.975	**0.003**
biomass	−0.4177	0.0666	223.23	−6.27	**<0.001**
Marginal R^2 / Conditional R^2: 0.13 / 0.63					

Supplementary Table 12. The path coefficients of structural equation models for organic fertilization.

Model: Species richness response ratio ~ SWC + SEC + SBD +MAT + TN + N +Org<br>Aboveground biomass response ratio ~ SWC + SCEC + SBD + MAT + TN + N + P + Org<br>Species richness response ratio ~~ Aboveground biomass response ratio				
Regressions:				
	Estimate	SE	*p* value	Std.lv
Species richness response ratio ~				
SWC	0.442	0.220	**0.045**	0.442
SBD	0.064	0.360	0.859	0.064
SCEC	0.069	0.207	0.739	0.069
MAT	−0.247	0.250	0.323	−0.247
TN	−0.847	0.335	**0.012**	−0.847
N	−0.230	0.179	0.199	−0.230
Org	0.321	0.306	0.295	0.321
Aboveground biomass response ratio ~				
SWC	−0.144	0.141	0.309	−0.144
SBD	−0.730	0.224	0.001	−0.730
SCEC	−0.141	0.126	0.266	−0.141
MAT	0.218	0.164	0.185	0.218
TN	−0.661	0.218	**0.002**	−0.661
N	0.377	0.108	**0.000**	0.377
P	0.245	0.102	**0.016**	0.245
Org	0.832	0.187	**0.000**	0.832
Covariances:				
Species richness response ratio ~~Aboveground biomass response ratio	−0.058	0.052	0.271	−0.058
Variances:				
Species richness response ratio	0.424	0.120	**0.000**	0.424
Aboveground biomass response ratio	0.154	0.044	**0.000**	0.154
Species richness response ratio R^2	0.558			
Aboveground biomass response ratio R^2	0.841			
Model parameter	χ^2 = 0.756, *df* = 1, *p* = 0.385, *RMSEA* = 0.000, *GFI* = 0.998, *CFI* = 1.000			

Note: χ^2: chi-square, *p*: *p* value, *df*: degree of freedom, *RMSEA*: root mean square error of approximation, *GFI*: comparative fit index, *CFI*: goodness of fit index indicating that the models fit reasonably when $p > 0.05$, $RMSEA \leq 0.08$, $GFI > 0.90$, $CFI > 0.95$. R^2

represents the proportion of variance explained for the dependent variable. The values in bold indicate a significant effect (two-sided, $p < 0.05$). See Table 3 for environmental factors. Source data are provided as a Source Data file.

Table 12 (continued). The path coefficients of structural equation model for inorganic fertilization

Model: Species richness response ratio ~ N + P + SWC<br>Above ground biomass response ratio ~ N + P + Niche + SWC<br>Species richness response ratio ~~ Above ground biomass response ratio				
Regressions:				
	Estimate	SE	*p* value	Std.lv
Species richness response ratio ~				
N	−0.425	0.050	**0.000**	−0.425
P	−0.538	0.050	**0.000**	−0.538
SWC	0.116	0.050	**0.022**	0.116
Above ground biomass response ratio ~				
N	0.259	0.059	**0.000**	0.259
P	0.022	0.068	0.750	0.022
Niche	0.374	0.067	0.000	0.374
SWC	−0.022	0.061	0.715	−0.022
Covariances:				
Species richness response ratio ~~Aboveground biomass response ratio	−0.088	0.043	**0.041**	−0.088
Variances:				
Species richness response ratio	0.550	0.051	**0.000**	0.550
Aboveground biomass response ratio	0.765	0.072	**0.000**	0.765
Species richness response ratio R^2	0.447			
Aboveground biomass response ratio R^2	0.232			
Model parameter	$\chi^2 = 0.007$, $df = 1$, $p = 0.935$, $RMSEA = 0.000$, $GFI = 1.000$, $CFI = 1.000$			

Note: When the number of nutrients added (Niche) was included in the model, the fitting coefficient test failed, therefore it was excluded in the final model. Source data are provided as a Source Data file.

We further collected data of quality (C:N ratios) of organic fertilizers, which were available in about 40% of all the organic studies. The C:N ratios of organic fertilizers were generally similar between grasslands and croplands, ranged from 7 to 72 and from 7 to 80, respectively (Figure 6**d**). We included C:N ratios in the linear mixed effect models and partial regression analysis. Under organic fertilization, grasslands with higher mean annual temperature, the amount and C:N ratios of organic input, fertilization duration, soil pH, sand content and water content had greater increase in SOC (Figure 6). However, the SOC response ratio to organic fertilization decreased with increasing C:N ratios of organic fertilizer inputs, soil sand content and water content in croplands. Collectively, in grasslands, SOC response ratio under organic fertilization increased with increasing mean annual temperature after accounting for the effects of soil properties, the amount and quality of organic fertilizer added (Figure 6). Therefore, in the future warmer climate, organic fertilization in grasslands may have larger soil carbon sequestration potential than in croplands.

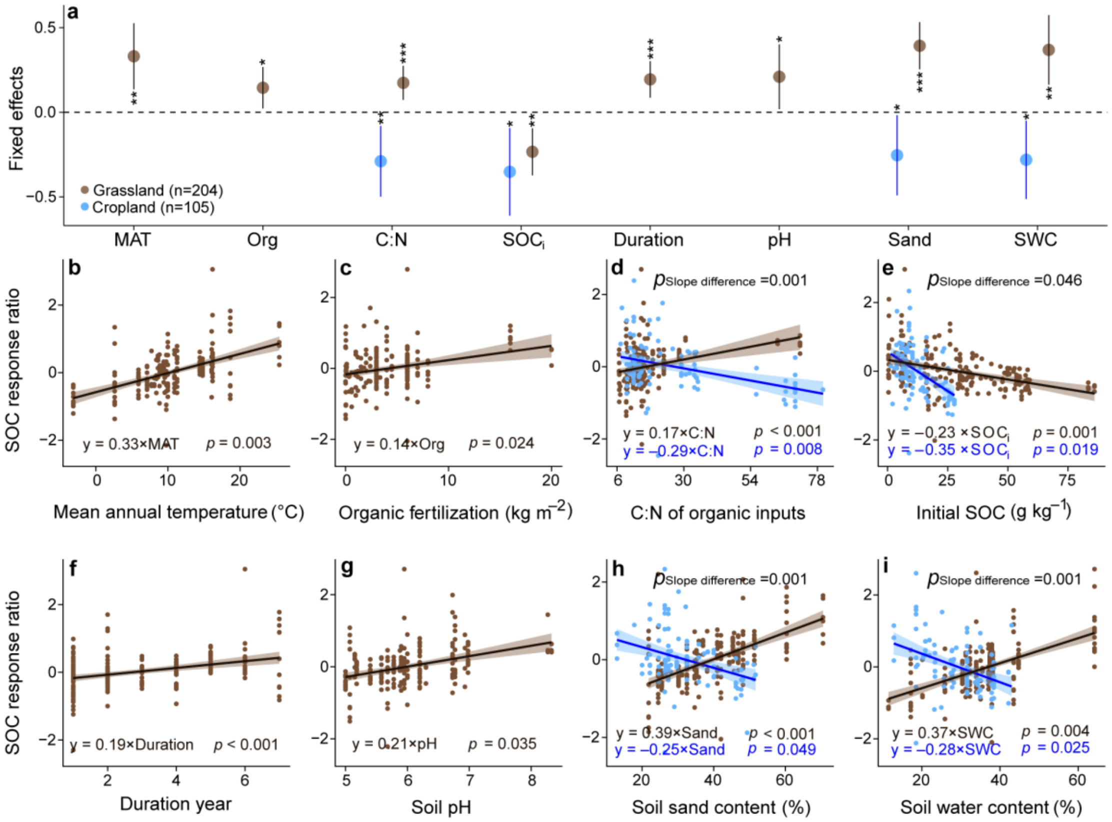

Supplementary Figure 6. The responses of soil organic carbon (SOC) to organic fertilization across environmental gradients in global grasslands and croplands.

Brown and blue represent grassland and cropland, respectively. We used linear mixed effects models to evaluate the impacts of climate, soil properties, the quantity and quality of organic inputs on SOC under organic fertilization. (**a**) Circles and error bars represent average parameter estimates (standardized regression coefficients) and 95% confidence intervals in the linear mixed effect models, respectively. The environmental factors were obtained from the best model selected based on Akaike Information Criterion (**b**–**i**). The brown and blue lines in each panel show model fits using partial regression for each environmental factor, and the brown and blue area around the fit lines represents the 95% confidence intervals. Equations in **b**–**i** show the values of standardized regression coefficients. The slopes of the partial regressions are the same as the fixed effects shown in Figure 6**a**, we compared the difference in slopes between grasslands and croplands using the standardized major axis tests. **Key**: MAT: mean annual temperature, Org: organic fertilizer amount added, C:N: quality of organic inputs, SOC_i: initial SOC, Duration: duration year, pH: soil pH, Sand: soil sand content, SWC: soil water content; *$p < 0.05$, ** $p < 0.01$ and *** $p < 0.001$. The *p*-values were calculated from two-tailed tests. Source data are provided as a Source Data file.

Supplementary Table 13. The test statistics of the Standardised Major Axis Tests and Routines (SMATR) with ordinary least squares regression technique for Figure 5**c**–**e**.

Groups	Test statistics	*p values*
Mean annual temperature	131.828	**0.001**
Organic fertilization	0.988	0.304
Initial SOC	24.016	**0.001**

Note: The values in bold indicate a significant effect ($p < 0.05$). Source data are provided as a Source Data file.

Supplementary Note. PRISMA 2020 Checklist

Section and Topic	Item #	Checklist item	Location where item is reported
**TITLE**			
Title	1	The report is identified as a meta-analysis. The title of this study is "A global meta-analysis on the effects of organic and inorganic fertilization on grasslands and croplands".	Page 1, line 1
**ABSTRACT**			
Abstract	2	A central role for nature-based solutions is to identify optimal management practices to address environmental challenges, including carbon sequestration and biodiversity conservation. Inorganic fertilization increases plant aboveground biomass but often causes a trade-off with plant diversity loss. It remains unclear, however, whether organic fertilization, as a potential nature-based solution, could alter this tradeoff by increasing aboveground biomass without plant diversity loss. Here we compile data from 537 experiments on organic and inorganic fertilization across grasslands and croplands worldwide to evaluate the responses of plant aboveground biomass, plant diversity and soil organic carbon (SOC). Both organic and inorganic fertilization increase aboveground biomass by 56% and 42% relative to ambient, respectively. However, only inorganic fertilization decreases plant diversity, while organic fertilization increases plant diversity in grasslands with greater soil water content. Moreover, organic fertilization increases SOC in grasslands by 19% and 15% relative to ambient and inorganic fertilization, respectively. The positive effect of organic fertilization on SOC increases with increasing mean annual temperature in grasslands, a pattern not observed in croplands. Collectively, our findings highlight organic fertilization as a potential nature-based solution that can increase two ecosystem services of grasslands, forage production and soil carbon storage, without a tradeoff in plant diversity loss.	Page 2, lines 13-31.
**INTRODUCTION**			
Rationale	3	A central role for nature-based solutions is to identify optimal management practices to address environmental challenges, including carbon sequestration and biodiversity conservation. Inorganic fertilization increases plant aboveground biomass but often causes a trade-off with plant diversity loss. It remains unclear, however, whether organic fertilization, as a potential nature-based solution, could alter this tradeoff by increasing aboveground biomass without plant diversity loss	Page 2, lines 13-18.
Objectives	4	We explore four main hypotheses: (1) organic fertilization would increase more aboveground biomass than did inorganic fertilization in grasslands, (2) if increased biomass production intensified competition for light, or fertilization reduced belowground niche partitioning, organic fertilization would also cause a decline in plant diversity in grasslands, and (3) if nitrogen detriment (e.g., acidification) was the main mechanism, organic fertilization would not cause plant diversity loss in grasslands. Finally, (4) we hypothesized that organic fertilizer added to croplands would lead to comparable increases in SOC compared to grasslands.	Page 6, lines 120-130.
**METHODS**			
Eligibility criteria	5	We used three criteria to select literature: (1) field experiments were conducted in semi-natural or natural grasslands, or croplands, and included both ambient and nutrient addition treatments; (2) the means, standard errors or standard deviations and sample sizes were reported; and (3) grassland studies reporting exotic plant species introduced by organic fertilization were excluded	Page 17, lines 390-394.
Information sources	6	Web of science and China National Knowledge Network resources.<br>We started collecting data in October 2022 and end in February 2023.	Page 17, line 379.

Section and Topic	Item #	Checklist item	Location where item is reported
Search strategy	7	We searched for peer-reviewed literature published before 30 October 2022 using the web of science and China National Knowledge Network resources. We used the following keywords: (resource addition OR resource availability OR nutrient addition OR nutrient availability OR nitrogen deposition OR nitrogen addition OR nitrogen enrichment OR phosphorus addition OR phosphorus enrichment OR potassium addition OR potassium enrichment OR organic fertilizer OR organic* OR manure* OR farmyard manure* OR pig manure OR cow manure OR horse manure OR sheep manure OR chicken manure OR wet compost) AND (species richness OR plant diversity OR biomass OR aboveground biomass OR AGB OR dry matter yield OR SOC OR soil organic carbon OR SOM OR soil organic matter OR SOC storage) AND (grassland OR meadow OR steppe OR prairie OR herbaceous OR annual OR cropland).	Page 17, lines 380-389.
Selection process	8	Studies identified from web of science and China National Knowledge Network resources: Databases (n=26163), Register (n=0).<br>Studies removed before screening: 1. Duplicate records were removed by using Endnote software (n=2258); 2. Records marked as ineligible by automation tools (15692). 3. Records removed for other reasons, i.e., review papers. 4. Records excluded after abstract and full text screening.<br>Reports excluded: 1. Studies belonged to laboratory cultivation experiments and artificial grasslands (n=2282). 2. Studies did not report the means, standard errors or standard deviations (n=1055). 3. Studies reported exotic plant species introduced by organic fertilization (n=8).	Supplementary Figure 1
Data collection process	9	Data were collected simultaneously by Ting-Shuai Shi and Hai-Ling Li from tables in the main text or supporting information when available, or digitally extracted from figures using GetData Graph Digitizer software version 2.26 (http://getdata-graph-digitizer.com/).	Page 17, lines 394-399.
Data items	10a	TS=(resource addition OR resource availability OR nutrient addition OR nutrient availability OR nitrogen deposition OR nitrogen addition OR nitrogen enrichment OR phosphorus addition OR phosphorus enrichment OR potassium addition OR potassium enrichment OR organic fertilizer OR organic* OR manure* OR farmyard manure* OR pig manure OR cow manure OR horse manure OR sheep manure OR chicken manure OR wet compost) AND (species richness OR plant diversity OR biomass OR aboveground biomass OR AGB OR dry matter yield OR SOC OR soil organic carbon OR SOM OR soil organic matter OR SOC storage) AND (grassland OR meadow OR steppe OR prairie OR herbaceous OR annual OR cropland).<br>We used three criteria to select literature: (1) field experiments were conducted in semi-natural or natural grasslands, or croplands, and included both ambient and nutrient addition treatments; (2) the means, standard errors or standard deviations and sample sizes were reported; and (3) grassland studies reporting exotic plant species introduced by organic fertilization were excluded	Page 17, lines 380-394.
	10b	Variable names: species richness, plant diversity, biomass, aboveground biomass, AGB, dry matter yield, SOC, soil organic carbon, SOM, soil organic matter and SOC storage. We directly excluded studies with unclear information	Page 17
Study risk of bias assessment	11	Egger’s regression test and funnel plot.	Supplementary Table1 and Figure 3
Effect measures	12	We used the natural log-transformed response ratio (ln RR=ln(Y_t/Y_c)) to quantify the effect of nutrient fertilization on aboveground biomass, plant diversity and SOC. The ln RR, also called “effect size”, was dimensionless and used to characterize the relative changes between treatment and control. Where "Y_t " and "Y_c" are sample mean values of the response variables (aboveground biomass, or plant diversity, or SOC) in the treatment group (t) and control group (c), respectively.	Pages 18-19, lines 425-432.

Section and Topic	Item #	Checklist item	Location where item is reported
Synthesis methods	13a	We used a hierarchical model (i.e., random effect model) with inverse variance weighting to summarize the response ratio (ln RR) from all individual studies, as this model was usually appropriate for biological experiments.	Page 19, line 436-438.
	13b	Because multiple treatments may share a single control, we added "site" as a random factor in the meta-analysis model to account for non-independence of observations collected from the same site.	Page 19
	13c	If you want to show the model results, you can use “summary” function in R to visualize the results.	See R codes for more details
	13d	We used a hierarchical model with inverse variance weighting to summarize the response ratio (ln RR) from all individual studies, as this model was usually appropriate for biological experiments. Because multiple treatments may share a single control, we added "site" as a random factor in the meta-analysis model to account for non-independence of observations collected from the same site.<br>Specifically, we used the "rma.mv" function in R "metafor" package version 4.4.0 to calculate the weighted mean response ratio (ln RR++) and the 95% confidence intervals. The 95% confidence intervals were generated by bootstrapping. When they did not overlap with zero, the treatment effects were considered statistically significant.	Page 19, lines 436-444.
	13e	We used linear mixed effects models to evaluate the response of biomass, plant diversity and SOC to nutrient fertilization across environmental gradients, with study site as a random effect. We conducted linear mixed models in "lme4" package version 1.1-35.1 and "lmerTest" packages version 3.1.3. To select the set of environmental factors that significantly influenced the response of biomass, plant diversity and SOC to nutrient addition, we conducted a multi-model inference procedure based on the Akaike Information Criterion, using the dredge function in R "MuMIn" package version 1.47.5. To further strengthen the multi-model inference, we also conducted a random forest model to identify the significant environmental predictors of biomass and plant diversity. We used random forest models in the "randomForest" package version 4.7-1.1 to quantify the importance of each predictor, and then used the "rfPermute" package version 2.5.2 to assess the statistical significance of each predictor.	Page 20, lines 465-477.
	13f	We applied the Egger’s test to examine publication bias, and used the trim and fill approach to evaluate the impact of publication bias on the meta-analysis results.	Supplementary Table 1 and Figure 3.
Reporting bias assessment	14	Egger’s regression test and funnel plot.	Page 19, Supplementary Table 1 and Figure 3.
Certainty assessment	15	We calculate the weighted mean response ratio (ln RR++) and the 95% confidence intervals. The 95% confidence intervals were generated by bootstrapping. When they did not overlap with zero, the treatment effects were considered statistically significant.	Page 19, 441-444.
**RESULTS**			
Study selection	16a	Studies identified from Web of science and China National Knowledge Network resources: Databases (n=26163) Registers (n=0)<br>Studies removed before screening:1. Duplicate records were removed by using Endnote software (n=2258). 2. Records marked as ineligible by automation tools (n=15692). 3. Records removed for other reasons, i.e., review papers (n=2118).	Supplementary Figure 1

Section and Topic	Item #	Checklist item	Location where item is reported
		Records excluded after abstract and full text screening: (n=2213)	
	16b	Reports excluded: Reason 1: studies belonged to laboratory cultivation experiments and artificial grasslands (n=2282). Reason 2: studies did not report the means, standard errors or standard deviations (n=1055). Reason 3: studies reported exotic plant species introduced by organic fertilization (n=8)	Supplementary Figure 1
Study characteristics	17	The variable names were extracted including: species richness, plant diversity, biomass, aboveground biomass, AGB, dry matter yield, SOC, soil organic carbon, SOM, soil organic matter and SOC storage. Moreover, we also extract the coordinate information for each study.	Source data files
Risk of bias in studies	18	Egger's regression test and funnel plot.	Supplementary Table 1 and Figure 3.
Results of individual studies	19	Values of weighted mean response ratio (ln RR++) and corresponding confidence intervals in the meta-analysis as shown in Supplementary Table 2.	Supplementary Table 2.
Results of syntheses	20a	Egger's regression suggested that there was potential publication bias in aboveground biomass, species richness, Pielou evenness index and soil organic carbon datasets under inorganic fertilization (Supplementary Table 1), while the trim and fill analysis suggested that there were no missing values (Supplementary Figs. 2e, f, g and h). In addition, we found a potential publication bias in aboveground biomass and species richness datasets under organic fertilization, and the trim and fill results are shown in Supplementary Figure 2a–b. The trim and fill analysis suggested that there was no missing value in species richness under organic fertilization (Supplementary Fig. 2b). There were some missing values for aboveground biomass, but we found that the meta-analysis results for aboveground biomass had little impact before and after trimming and filling (before: ln RR++ = 0.37, $p<0.001$; after: ln RR++ = 0.19, $p<0.001$). Therefore, our meta-analysis results were robust and reliable.	Supplementary Figure 3.
	20b	Please see Supplementary Table 1 and Table 2.	Supplementary information
	20c	Present results of all investigations of possible causes of heterogeneity among study results	NA
	20d	Present results of all sensitivity analyses conducted to assess the robustness of the synthesized results.	Supplementary Table 1 and Figure 3.
Reporting biases	21	Egger's regression suggested that there was potential publication bias in aboveground biomass, species richness, Pielou evenness index and soil organic carbon datasets under inorganic fertilization (Table 1), while the trim and fill analysis suggested that there were no missing values (Supplementary Figs. 2e, f, g and h). In addition, we found a potential publication bias in aboveground biomass and species richness datasets under organic fertilization, and the trim and fill results are shown in Supplementary Figure 2a–b. The trim and fill analysis suggested that there was no missing value in species richness under organic fertilization (Supplementary Fig. 2b). There were some missing values for aboveground biomass, but we found that the meta-analysis results for aboveground biomass had little impact before and after trimming and filling (before: ln RR++ = 0.37,	Supplementary Figure 3.

Section and Topic	Item #	Checklist item	Location where item is reported
		p<0.001; after: ln RR++ = 0.19, *p*<0.001).	
Certainty of evidence	22	We applied the Egger's test to examine publication bias, and used the trim and fill approach to evaluate the impact of publication bias on the meta-analysis results. The test results show our meta-analysis results were robust and reliable. See Supplementary Table 1 and Figure 3 for more details.	Supplementary Table 1 and Figure 3.
**DISCUSSION**			
Discussion	23a	We have provided a general interpretation of the results.	Pages 13-16
	23b	Discuss any limitations of the evidence included in the review.	NA
	23c	Discuss any limitations of the review processes used.	NA
	23d	We argue that increasing the use of organic fertilizers would provide an important nature-based solution to increase productivity and soil carbon sequestration while conserving plant diversity.	Page 16
**OTHER INFORMATION**			
Registration and protocol	24a	The protocol is described in the Methods, Registration does not apply.	NA
	24b	Indicate where the review protocol can be accessed, or state that a protocol was not prepared.	NA
	24c	Describe and explain any amendments to information provided at registration or in the protocol.	NA
Support	25	Describe sources of financial or non-financial support for the review, and the role of the funders or sponsors in the review.	NA
Competing interests	26	The authors declare no competing interests.	Page 26
Availability of data, code and other materials	27	All data and code used in this study, including raw data and source data underlying figures, has been deposited in Figshare (https://doi.org/10.6084/m9.figshare.25493419). Mean annual temperature at each site was extracted from the WorldClim database (https://www.worldclim.org/). Soil cation exchange capacity, total nitrogen, pH, bulk density, organic carbon density and sand content were extracted from Soil Grid database (https://files.isric.org/soilgrids/latest/data_aggregated/1000m/). Soil water content was obtained from ERA5-Land database (https://www.ecmwf.int/en/era5-land). Global map was downloaded from natural earth (https://www.naturalearthdata.com/).	Page 21, lines 492-500

From: Page MJ, McKenzie JE, Bossuyt PM, Boutron I, Hoffmann TC, Mulrow CD, et al. The PRISMA 2020 statement: an updated guideline for reporting systematic reviews. BMJ 2021;372:n71. doi: 10.1136/bmj.n71

For more information, visit: http://www.prisma-statement.org/
