# Supplementary material for: A global meta-analysis on the effects of organic and inorganic fertilization on grasslands and croplands
Source: Nat Commun. 2024 Apr 22;15:3411. doi: 10.1038/s41467-024-47829-w (PMC11035549; doi:10.1038/s41467-024-47829-w)
Supplement: Supplementary file 2 — Reporting Summary [file 41467_2024_47829_MOESM2_ESM.pdf]

nature portfolio

Corresponding author(s): Jian-Sheng Ye
Last updated by author(s): Apr 11, 2024

Reporting Summary

Nature Portfolio wishes to improve the reproducibility of the work that we publish. This form provides structure for consistency and transparency in reporting. For further information on Nature Portfolio policies, see our Editorial Policies and the Editorial Policy Checklist.

Statistics

For all statistical analyses, confirm that the following items are present in the figure legend, table legend, main text, or Methods section.

n/a	Confirmed	
☐	☒	The exact sample size (*n*) for each experimental group/condition, given as a discrete number and unit of measurement
☐	☒	A statement on whether measurements were taken from distinct samples or whether the same sample was measured repeatedly
☐	☒	The statistical test(s) used AND whether they are one- or two-sided<br>*Only common tests should be described solely by name; describe more complex techniques in the Methods section.*
☐	☒	A description of all covariates tested
☐	☒	A description of any assumptions or corrections, such as tests of normality and adjustment for multiple comparisons
☐	☒	A full description of the statistical parameters including central tendency (e.g. means) or other basic estimates (e.g. regression coefficient) AND variation (e.g. standard deviation) or associated estimates of uncertainty (e.g. confidence intervals)
☐	☒	For null hypothesis testing, the test statistic (e.g. *F*, *t*, *r*) with confidence intervals, effect sizes, degrees of freedom and *P* value noted<br>*Give P values as exact values whenever suitable.*
☒	☐	For Bayesian analysis, information on the choice of priors and Markov chain Monte Carlo settings
☐	☒	For hierarchical and complex designs, identification of the appropriate level for tests and full reporting of outcomes
☒	☐	Estimates of effect sizes (e.g. Cohen's *d*, Pearson's *r*), indicating how they were calculated

Our web collection on statistics for biologists contains articles on many of the points above.

Software and code

Policy information about availability of computer code

Data collection	To compile data on the responses of plant biomass, plant diversity and SOC to nutrient addition, we searched for peer-reviewed literature published before 30 October 2022 using the web of science and China National Knowledge Network resources. We used the following keywords: (resource addition OR resource availability OR nutrient addition OR nutrient availability OR nitrogen deposition OR nitrogen addition OR nitrogen enrichment OR phosphorus addition OR phosphorus enrichment OR potassium addition OR potassium enrichment OR organic fertilizer OR organic* OR manure* OR farmyard manure* OR pig manure OR cow manure OR horse manure OR sheep manure OR chicken manure OR wet compost) AND (species richness OR plant diversity OR biomass OR aboveground biomass OR AGB OR dry matter yield OR SOC OR soil organic carbon OR SOM OR soil organic matter OR SOC storage) AND (grassland OR meadow OR steppe OR prairie OR herbaceous OR annual OR cropland).<br>Then, we collected data from from tables in main text or supporting information when available, or digitally extracted from figures using GetData Graph Digitizer version 2.22.
Data analysis	We conducted statistical analyses using "lme4" package(version 4.4.0), "lmerTest" package(version 3.1.3), "MuMIn" package(1.47.5), "rfPermute" package (version 2.5.2), "lavaan" package(version 0.6-17),"randomForest" package (version 4.7-1.1) in R 4.3.2. The associated analysis code is archived on figure (https://doi.org/10.6084/m9.figshare.25493419). Figures were produced in R and polished in Adobe Illustrators (2021).

For manuscripts utilizing custom algorithms or software that are central to the research but not yet described in published literature, software must be made available to editors and reviewers. We strongly encourage code deposition in a community repository (e.g. GitHub). See the Nature Portfolio guidelines for submitting code & software for further information.

Data

Policy information about availability of data

All manuscripts must include a data availability statement. This statement should provide the following information, where applicable:

- Accession codes, unique identifiers, or web links for publicly available datasets
- A description of any restrictions on data availability
- For clinical datasets or third party data, please ensure that the statement adheres to our policy

All data used in this study, including raw data and source data underlying figures, has been deposited in Figshare(https://doi.org/10.6084/m9.figshare.25493419). Mean annual temperature at each site was extracted from the WorldClim database (https://www.worldclim.org/). Soil cation exchange capacity, total nitrogen, pH, bulk density, organic carbon density and sand content were extracted from Soil Grid database (https://files.isric.org/soilgrids/latest/data_aggregated/1000m/). Soil water content was obtained from ERA5-Land database (https://www.ecmwf.int/en/era5-land). Global map was downloaded from natural earth (https://www.naturalearthdata.com/). Source data are provided with this paper.

Research involving human participants, their data, or biological material

Policy information about studies with human participants or human data. See also policy information about sex, gender (identity/presentation), and sexual orientation and race, ethnicity and racism.

Reporting on sex and gender	Not application. Our research does not involve human participants, their data, or biological material.
Reporting on race, ethnicity, or other socially relevant groupings	Not application. Our research does not involve human participants, their data, or biological material.
Population characteristics	Not application. Our research does not involve human participants, their data, or biological material.
Recruitment	Not application. Our research does not involve human participants, their data, or biological material.
Ethics oversight	Not application. Our research does not involve human participants, their data, or biological material.

Note that full information on the approval of the study protocol must also be provided in the manuscript.

Field-specific reporting

Please select the one below that is the best fit for your research. If you are not sure, read the appropriate sections before making your selection.

☐ Life sciences ☐ Behavioural & social sciences ☒ Ecological, evolutionary & environmental sciences

For a reference copy of the document with all sections, see nature.com/documents/nr-reporting-summary-flat.pdf

Ecological, evolutionary & environmental sciences study design

All studies must disclose on these points even when the disclosure is negative.

Study description

In this study, we compiled data from 537 experiments on organic and inorganic fertilization across grasslands and croplands worldwide to evaluate their impacts on aboveground biomass, plant diversity and soil carbon storage.

Moreover, we hypothesized that: (1) organic fertilization would increase more aboveground biomass than did inorganic fertilization in grasslands, (2) if increased biomass production intensified competition for light, or fertilization reduced belowground niche partitioning, organic fertilization would also cause a decline in plant diversity in grasslands, and (3) if nitrogen detriment (e.g., acidification) was the main mechanism, organic fertilization would not cause plant diversity loss in grasslands. We also evaluated the effect of fertilization on biomass,plant diversity and SOC across environmental gradients to determine under which conditions tradeoffs among the three ecosystem services would be minimized. Finally, (4) we hypothesized that organic fertilizer added to croplands would lead to comparable increases in SOC compared to grasslands.

Research sample

For the meta-analysis study, we compiled 1540 pairs (ambient versus fertilization) of field measurements of aboveground biomass, 1625 pairs of species richness, 191 pairs of Pielou evenness index, and 799 pairs of soil organic carbon content (SOC) under inorganic fertilization. Under organic fertilization, we compiled 350 pairs of aboveground biomass, 155 pairs of species richness, 89 pairs of Pielou evenness index, 388 pairs of SOC and 367 pairs of cropland SOC.

To prevent bias in publication selection, we use three criteria to select the most appropriate studies: (1) field experiments were conducted in semi-natural or natural grasslands, or croplands, and included both ambient and nutrient addition treatments; (2) the means, standard errors or standard deviations and sample sizes were reported; and (3) grassland studies reporting exotic plant species introduced by organic fertilization were excluded.

Furthermore, this meta-analysis study also involve use of existing datasets including WorldClim database (https://

	www.worldclim.org/), the Soil Grid database (https://files.isric.org/soilgrids/latest/data_aggregated/1000m/), the ERA5-Land database (https://www.ecmwf.int/en/era5-land) and the Global map (https://www.naturalearthdata.com/).
Sampling strategy	We used three criteria to select literature: (1) field experiments were conducted in semi-natural or natural grasslands, and included both ambient and nutrient addition treatments; (2) the means, standard errors or standard deviations and sample sizes were reported; and (3) studies reporting exotic plant species introduced by organic fertilization were excluded.
Data collection	Data were collected by TingShuai Shi and HaiLing Li from tables in the main text or supporting information when available, or digitally extracted from figures using GetData Graph Digitizer software version 2.26 (http://getdata-graph-digitizer.com/). The PRISMA flow diagram (Supplementary Figure 1) showed the procedure we used for selection of studies.<br>Moreover, Tingshuai Shi downloads and extracts climate and soil data.
Timing and spatial scale	Timing scale: data published before 2023, as early as data published in 1965.<br>Spatial scale: global grasslands and croplands, excluding forest.<br>We started collecting data in October 2022 and end in February 2023. Specifically, we strictly had to complete 20% of the workload each month, which mainly data collection and extraction.
Data exclusions	We carefully excluded experiments where organic fertilizer introduced exotic plant seeds.
Reproducibility	As our study is a meta-analysis, we did not perform an experiment. The literature search conducted in the WoS database is fully reproducible. For screening of the eligible papers, we set clear criteria for inclusion and exclusion that help reproducibility (see Methods section). Every step of statistical analysis is described.<br>All raw data are deposited in Figshare: https://doi.org/10.6084/m9.figshare.25493419.
Randomization	Samples were grouped by locations and ecosystem properties, as described in detail in the Methods section.
Blinding	We are blinded to group allocation mainly through two ways. Firstly, we set explicit rules of including a study before compiling the data from the published studies, as stated clearly in the Method section, Secondly, our database included all data in the accessible and published studies that fulfilled our rules of including an experiment, without the exclusion of any of experiments for the statistical analyses

Did the study involve field work? ☐ Yes ☒ No

Reporting for specific materials, systems and methods

We require information from authors about some types of materials, experimental systems and methods used in many studies. Here, indicate whether each material, system or method listed is relevant to your study. If you are not sure if a list item applies to your research, read the appropriate section before selecting a response.

Materials & experimental systems

n/a	Involved in the study
☒	☐ Antibodies
☒	☐ Eukaryotic cell lines
☒	☐ Palaeontology and archaeology
☒	☐ Animals and other organisms
☒	☐ Clinical data
☒	☐ Dual use research of concern
☒	☐ Plants

Methods

n/a	Involved in the study
☒	☐ ChIP-seq
☒	☐ Flow cytometry
☒	☐ MRI-based neuroimaging

Plants

Seed stocks	Not application. Our research does not involve seed stocks.
Novel plant genotypes	Not application. Our research does not involve novel plant genotypes.
Authentication	Not application. Our research does not involve authentication.
