# Supplementary material for: A global meta-analysis on the effects of organic and inorganic fertilization on grasslands and croplands
Source: Nat Commun. 2024 Apr 22;15:3411. doi: 10.1038/s41467-024-47829-w (PMC11035549; doi:10.1038/s41467-024-47829-w)
Supplement: Supplementary file 3 — Description of Additional Supplementary Files [file 41467_2024_47829_MOESM3_ESM.pdf]

File Name: Supplementary Data 1

Description: Datasets of inorganic (Sheet 1) and organic (Sheet 2) fertilization compiled in this meta-analysis.
